# Impact of cholesterol-pathways on breast cancer development, a metabolic landscape

**DOI:** 10.7150/jca.54637

**Published:** 2021-05-19

**Authors:** Alina González-Ortiz, Octavio Galindo-Hernández, Gerson N. Hernández-Acevedo, Gustavo Hurtado-Ureta, Victor García-González

**Affiliations:** Departamento de Bioquímica, Facultad de Medicina Mexicali, Universidad Autónoma de Baja California, 21000 Mexicali, México.

**Keywords:** breast cancer, plasmatic lipoproteins, SREBP-2/mevalonate pathway, oxysterols, epoxy-cholesterols

## Abstract

ApoB-lipoproteins and their components modulate intracellular metabolism and have been associated with the development of neoplastic phenomena, such as proliferation, anchorage-independent growth, epithelial-mesenchymal transition, and cancer invasion. In cancer cells, the modulation of targets that regulate cholesterol metabolism, such as synthesis *de novo*, endocytosis, and oxidation, are contributing factors to cancer development. While mechanisms associated with sterol regulatory element-binding protein 2 (SREBP-2)/mevalonate, the low-density lipoprotein receptor (LDL-R) and liver X receptor (LXR) have been linked with tumor growth; metabolites derived from cholesterol-oxidation, such as oxysterols and epoxy-cholesterols, also have been described as tumor processes-inducers. From this notion, we perform an analysis of the role of lipoproteins, their association with intracellular cholesterol metabolism, and the impact of these conditions on breast cancer development, mechanisms that can be shared during atherogenesis promoted mainly by LDL. Pathways connecting plasma dyslipidemias in conjunction with the effect of cholesterol-derived metabolites on intracellular mechanisms and cellular plasticity phenomena could provide new approaches to elucidate the triggering factors of carcinogenesis, conditions that could be considered in the development of new therapeutic approaches.

## Introduction

**Figure 1:**
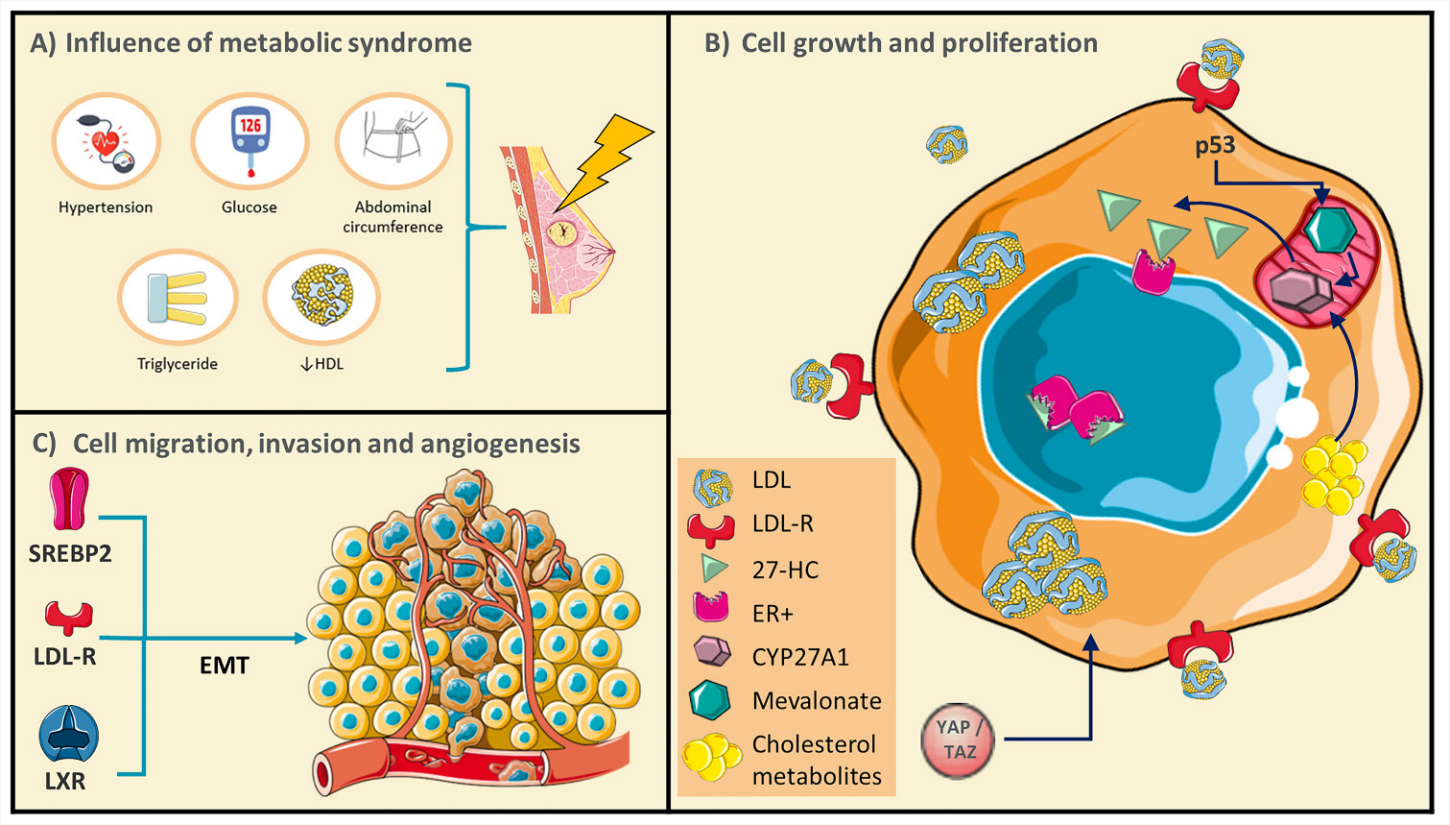
** Conditions of metabolic syndrome (MetS) could generate a favorable microenvironment for breast cancer (BC) development. A)** Schematic representations of MetS conditions. **B)** BC cells undergo rearrangements in the pathways of cholesterol metabolism, and several cholesterol derived-metabolites have been described as promoters of cellular growth and proliferation. **C)** Targets that control cholesterol metabolism could promote BC cell migration and invasion through epithelial-mesenchymal transitions (EMT). Adapted from: 21, 24, 25, 32, 48, 49.

Metabolic syndrome (MetS) is a pathological condition characterized by the presence of least three of five of the following medical conditions: abdominal obesity, elevated blood pressure, elevated fasting plasma glucose, high serum triglycerides, and low High-density lipoprotein (HDL) concentrations, therefore a dyshomeostasis of plasmatic lipids is present (Figure 1) 1-6. Specifically, abdominal obesity could be associated with insulin resistance and the development of dyslipidemias such as high LDL (low-density lipoprotein), low HDL concentrations, hyperglycemia and, consequently type 2 diabetes (T2D). Although its role in breast cancer (BC) is not fully elucidated, evidence supports that MetS could increase the risk of BC, and once diagnosed, impacts the prognosis of the disease. In a pioneer study, the Apolipoprotein Mortality Risk (AMORIS) comprised 812,073 Swedish individuals, compared lipid profiles from BC patients, measured by 8 years previous to diagnosis, results showed weak evidence associated with impaired triglycerides concentrations 7. Likewise, in a report of 208 BC patients versus a control group of 176 healthy women, patients with lymph node metastasis showed plasmatic altered metabolites such as glucose, triglycerides, elevated LDL levels, as well as low HDL 8. However, the relationship between plasmatic dyslipidemias and intracellular lipid metabolism, and their implications on BC progression is not fully clear. Nowadays, there is increasing evidence of the role of lipid metabolism alterations as biomarkers of cancer prognosis and survival 9. Changes of expression of lipid-metabolism genes are found in several tumors, considering lipid metabolism modulates cellular processes range from plasmatic and organelle membrane organization and plasticity 10, 11, ATP production 12, to intracellular signaling alterations 13, 14.

Cancer growth and proliferation have been associated with oncogene activation or loss of tumor suppressor function; nevertheless, alteration of metabolic pathways would play critical roles, enabling cancer cells to acquire and synthesize nutrients, then counteract nutrient deficit. Moreover, obesity and dyslipidemias have been considered triggering conditions of neoplastic pathology, contributing to the increase of mortality rate 15. Interestingly, BC-cells express high levels of enzymes involved in *De novo* cholesterol synthesis and the treatment with statins could inhibit their proliferation in secondary tumors 16. These studies are not conclusive since evidence indicates that the underlying mechanisms are more complex. For instance, in the Nurses' Health Study (NHS) cohort (N=79,518), postmenopausal participants without a cancer history were followed for 12 years, statin treatment was not associated with risk of invasive BC, independent of histologic subtype 17. Indeed, in a case-control study of long-term, statin treatment was associated with increased risk of both 916 invasive ductal carcinomas (IDC) and 1,068 invasive lobular carcinomas (ILC); nonetheless of those tumor characteristics, only 6% of patients were treated with statins for more 10 years showing odds ratio (OR) of 1.83 and 1.97, respectively. The authors did not consider the association among the use of long-term statin, and BC risk diverges by estrogen receptor (ER) status, given the relatively small number of cases 18. Furthermore, an association has been proposed among the increase in the time of BC recurrence and the early administration of lipophilic statins as a cholesterol-LDL lowering therapy 19. Therefore, evaluation of this condition in other populations for a longer period is required. Even more, the metabolic reprogramming of tumors could be controlled by multiple mechanisms such as epigenetic factors that regulate gene expression and, critically the role of cholesterol-derived metabolites, phenomena analyzed in the following chapters.

## Breast cancer and MetS association

High levels of circulating and local estrogens, altered adipokine-concentrations, disrupted insulin signaling, modifications within the microbiome, and local and systemic effects of inflammation could contribute to the obesity and cancer association 20. Several reports suggested an increased risk of recurrence in obese *versus* normal weight women with BC diagnosed 21. Obesity could be the critical pathology which allows to explain the relationship among chronic low-grade inflammatory condition and the metabolism alterations during MetS 6, 9. For instance, in early stages, adipocyte hyperplasia has been shown to generate a low-oxygen environment, leading to the expression of inducible Hypoxia factor 1 alpha (HIF1α), resulting in processes such as infiltration of both tumor-associated neutrophils (TAN) and tumor associated macrophages (TAMs), development of epithelial-mesenchymal transitions (EMTs), as well as critically modifications in lipid metabolism and inflammation 9, 15, 20. Hypoxia and the increased nutrient-demand promote the death of adipocytes surrounded by macrophages generating Crown-like structures, these conditions lead to the release of free fatty acids (FFAs), activating pro-inflammatory pathways through Toll-like receptor 4.

Obesity is closely linked with the presence of metabolic disorders. Excess fat triggers extensive remodeling of the adipose tissue microenvironment in terms of its size, vascularity, and cellular and matrix composition; which is accompanied by deregulated secretion of adipose cytokines or so-called adipokines. In this sense, the increase in pro-inflammatory adipokines such as TNF-α, interleukin-6 (IL-6), IL-1, IL-8 and resistin has been associated with insulin resistance and potentially T2D 22. In turn, this condition denominated meta-inflammation could be associated with the development of BC 23. The relationship between obesity and insulin concentrations has been documented, as a feature of the pathophysiology of T2D.

Although, a direct connection with cancer development is not conclusive; the presence of obesity before diagnosis, insulin resistance during diagnosis, and hyperinsulinemia after treatment have been associated with an increased risk of mortality and recurrence of BC 24, 25. Data of 94,555 women free of cancer at baseline in the NIH-American Association of Retired Persons Diet and Health Study cohort (NIH-AARP), suggested individual MetS components, such as waist circumference, high cholesterol, and hypertension, were independently associated with an increased risk of BC mortality 5. In another report, components of MetS mainly obesity were associated with an increased risk of BC-recurrence 4. At this respect, in a retrospective cohort study, involving 4,216 women between the period of 1990 and 2008, 26 % of women had three components of MetS, and compared with MetS components-free women, the presence of ≥3 MetS components was associated with a modest risk of BC-deaths (Figure 1) 4. Likewise, in the analysis of 116 datasets of 43 articles included 38,940 cases of several cancers, 9,643 BC cases were identified; in 5,161 women the presence of MetS was associated with breast postmenopausal cancer 26. Even more, evidence suggests that the risk associated with various components of MetS may be synergistic, considering that the biological mechanisms that connect MetS components effects and breast cancer risk could share signaling pathways 27.

Nevertheless, among 8,641 women with local or regional invasive BC, waist circumference was associated with a higher risk of cardiovascular disease, although a direct association between BC and the risk of cardiovascular disease was not found 28. In another report, obesity was associated with an increased risk of BC-recurrence 4. For instance, MetS is 1.5 times more prevalent in Hispanic individuals compared with non-Hispanic whites or blacks 29. Hispanic BC-survivors possibly show less favorable metabolic profiles compared with non-Hispanic, and activities such as clinical practice interventions may attenuate these disappointments 29. Even more, in an observational study involving 86 women (included 46 premenopausal and 40 postmenopausal) with early-stage BC and diagnosed MetS-free, after completing (neo) adjuvant chemotherapy (12-week to 18-week), all MetS components and the overall MetS score were significantly increased after treatment 30. Therefore, the correlation between the presence of MetS components and the risk of BC is not conclusive, consequently, evaluation of conditions such as sample size, population and the status of patients should be considered.

## Role of serum lipoproteins

Alterations in cholesterol pathways such as *de novo* synthesis, cell internalization, and cholesterol transport mediated by plasmatic lipoproteins could regulate several mechanisms in the development of BC 31. Very low density lipoproteins (VLDL) and LDL could be critical in several mechanisms of tumor progression 32. For instance, the treatment of cancer cells with VLDL and LDL induces pro-tumoral processes such as proliferation, anchorage independent growth, development of EMTs, angiogenesis, and metastasis. Conversely, HDL inhibits mammosphere-formation and increases the sensitivity to radiation in mammary tumoral cells 33. Therefore, the lipid and protein content of plasmatic lipoprotein, and alterations on intracellular cholesterol pathways in breast tissues could perform important features on phenomena associated with BC-development.

### Protective mechanisms of HDL

In epidemiological studies, broad experimental evidence has shown low-HDL concentrations are connected with an increased risk of coronary artery disease 34. The cardioprotective effect of HDL and its main protein component, apo-A1 are related with the reverse transport of cholesterol from the artery wall to the liver 35, 36, a condition which has situated HDLs for a long time as a promissory target for cardiovascular pathologies treatment 37. In an important way, evidence suggests high levels of HDL are associated with a low risk of BC. In African-American women, an elevation of BC risk was associated with low HDL levels 38. Likewise, in Taiwanese women low HDL concentrations were related with a three-fold increase in the risk of BC 39. A further study compared lipid profiles of 208 BC-patients newly diagnosed without treatment, showing lower HDL levels in all stages with a diminution associated to advanced stages, whereas LDL levels were maintained elevated in every stage of BC 8.

Revision of 7,575 females during 13 years in Atherosclerosis Risk in Communities Cohort Study, suggested a positive association among low HDL in premenopausal women and an increased risk of BC 40. In a meta-analysis of 26 studies, involucrated 24,655 individuals with cancer, patients with higher HDL had a 37% reduced risk of death compared to patients with low HDL levels 41. In another report, in women newly diagnosed with invasive BC stage I/II, an inverse association was reported between cholesterol-HDL content, phospholipids, apolipoprotein-A1, and Ki67, a canonical proliferative marker 42. Likewise, in a multicenter case-control study (N=690/1380), higher HDL levels-associated with reduced risk of premenopausal BC were reported, specifically HDL concentrations > 60 mg/dL decreased BC risk by 0.49-fold compared to HDL < 50 mg/dL 43. In the characterization of the lipoprotein subfractions in BC patients (N= 56), Flote *et al* 42 reported a positive association between ApoA-1, HDL, and larger HDL subfractions, with expression of progesterone receptors, a marker of a good prognosis 42. Notwithstanding, systematic review and meta-analysis of prospective studies suggested a slight inverse association between HDL and the BC risk 44. In another report, the characterization of 1044 patients as a preoperative study in all BC stages according to TNM classification, demonstrated normal HDL-values in most stages 45. Therefore, several reports are contradictory, and possible is critical to consider the sample size, the cancer stage and HDL concentrations.

Although the protective role of HDL in BC have been described, in conditions such as MetS, diabetes and obesity, quality of HDL could be modified, for instance, in the characterization of healthy (N = 65) and T2D patients (N = 71), diabetic HDL showed higher levels of glycation and oxidation, with 250 and 127% increments, respectively 46. These HDL have been proposed as a risk factor related with proliferation, migration, and metastasis in BC models 46. Other authors have found in patients with breast, colorectal and lung cancer, a decreased activity of paraoxonase, an enzyme that binds HDL and is associated with anti-inflammatory and anti-oxidant properties 47.

### LDL associated mechanisms

LDL cholesterol is one of the main risk factors for cardiovascular disease 50, evidence suggests elevated serum LDL concentrations could be a contributing factor to increase recurrence and mortality in obese women with BC 50, 51. Indeed, through univariate association studies is reported that patients with higher levels of LDL at diagnosis showed large tumors, with higher differentiation grade, frequently express HER-2 and are diagnosed in more advanced stages 52. Plasmatic LDL concentrations greater than 144 mg/dL in BC patients are associated with high histological grade, high proliferative tumors, accompanied by a risk of lympho-vascular invasion and lymph node metastasis 52.

In the analysis of 1054 BC patients compared with 2483 healthy women stratified by age, Li *et al* characterized the lipid profile status at the beginning of diagnosis and during chemotherapy, lipid profiles become worsened after chemotherapy, increasing concentrations of total cholesterol, triglycerides, LDL, and apolipoprotein B; while levels of HDL and Apo-A1 decreased compared with pre-chemotherapeutic status 53. In another report, during chemotherapy of 394 BC-patients, triglycerides, LDL and apolipoprotein-B increased significantly 53. Although in a small study of cases (N=30) and controls (N=100), LDL concentrations were not modified 54. In The Malmo Diet and Cancer Study, which enrolled 17,035 women (1991-1996), BC risk was inversely associated with Apo-B concentrations 55, which is the main protein of VLDL and LDL. In an important way, in a Mendelian randomization of the effects of serum lipids and BC risk from genetic datasets of the Global Lipid Genetics Consortium and the Breast Cancer Association Consortium 56 involucrated >400,000 participants, Nowak and Ärnlöv (2018) reported that genetically raised LDL-cholesterol is associated with higher BC risk 56. The aforementioned data indicate the fundamental role of lipoprotein metabolism in response to chemotherapy, therefore describing the mechanisms involved will allow the identification of drug-targets and the design of novel therapies in BC patients. For instance, in cohort studies, under hormonal treatment or chemotherapy, patients who presented an overexpression of LDL-R showed a reduced recurrence-free survival 48. In this case, a novel possibility is the design of strategies that could modulate the expression of LDL-R on tumoral tissues, it is recommendable taking into account the *know-how* of the novel treatments directed to PCSK-9, based on monoclonal antibodies 57 and RNAi 58 used in cardiovascular-risk patients.

In a complementary way, in hypercholesterolemic murine models, tumors were consistently larger, high proliferative, and showed an increased capacity to metastasize to the lung 59, 60. Specifically, cancer cells treatment with elevated LDL concentrations overexpressed LDL-receptor (LDL-R), and induced larger size tumor compared to cell lines with basal expression. Indeed, LDL-R silencing in tumor cells induced a reduction in tumor size. In line with this notion, high expression of LDL-R in BC has been associated with decreased recurrence-free survival, predominantly in patients treated with systemic therapies 48. The relevance of LDL-R on triple-negative growth and HER2 tumors has been highlighted 48. Additional to the increase in the expression of LDL-R in several models, and then, in the promotion of LDL-endocytosis, the inhibition of acetyl-coenzyme A acetyltransferase (ACAT), responsible of cholesterol esterification in the cytoplasm, represents a biomarker in the regulation of intracellular cholesterol storage, and there is an implication on migration process, activating the Ras/Raf-1/MEK/ERK pathway 61. This mechanism promotes over-expression of the Slug transcription factor, which induces an increase in the expression of phospholipase D2 (PLD2) and the formation of β-catenin-T-cell factor (TCF)-4 transcription complexes. Importantly, PLD2 has been associated with metastasis, an increase in tumor size, and a poor prognosis 62.

Currently, molecular mechanisms associated with the pro-tumoral function of LDL have been described. Characterization on *in vitro* and *in vivo* models of BC revealed a decrease in cell adhesion and an increase in cell migration induced by LDL, corroborated by a down-regulation of the expression of adhesion molecules such as member 3 of the cadherin-related family, CD22, claudin and occludin. Besides, the LDL-induced proliferative effect dependent on the activation of the Akt and ERK pathways; have been reported, and these mechanisms are characteristic of the EMT process 63. Likewise, the treatment of L1 and L5 subfractions of LDL and VLDL increased the levels of mesenchymal markers Slug, vimentin and β-catenin, promoting migration and invasion of BC-cells, distinctive events of cells under the EMT process 32 (Figure 1). EMTs are originated in epithelial cells, which acquire mesenchymal characteristics through cytoskeleton reorganization, loss of basal-apical polarity, loss of cell-cell interactions, an increase in the secretion of metalloproteinases, promoting migratory and invasive capacity, accompanied by a fibroblastoid phenotype 64. During this cellular phenomenon, cancer cells temporarily acquire the ability to degrade the basement membrane, invade the surrounding tissue, and perform intravasation 64. Then, L1 and L5 subfractions could enhance the secretion of angiogenic factors in BC-cells and promote the formation of new blood vessels, resulting in a high tumor burden and a poor prognosis 32. Notwithstanding, extensive evidence is needed to confirm the role of LDL-R overexpression in tumors, even more, the characterization of pathways that regulate the intracellular accumulation of cholesterol. For this reason, the management of plasmatic LDL-cholesterol levels as a regulator of BC-progression is highly promising, especially due to the availability of low-cost drugs such as statins. However, in clinical studies there are still disparities in the results obtained with the use of statins, with variations dependent of the type of statin 19, 65. Therefore, this therapeutic vision must be subjected to prolonged analysis and determine the response of the different tumor phenotypes to the treatment of these drugs.

In addition, a positive correlation of higher VLDL concentrations and lymph node metastasis has been reported in BC patients, these lipoproteins are associated with the transport of cholesterol, triglycerides and metabolites such as oxysterols from liver to the tumor cells 42. Elevated levels of VLDL have been associated with enhanced VEGF-signaling, promoting lymphatic angiogenesis, and coupled with TAMs through inflammatory cascades, could contribute to nodal metastasis disease 42. Therefore, the exacerbated concentrations of cholesterol should promote alterations in the mechanisms that regulate its homeostasis, including alterations in *de novo* synthesis, endocytosis, modifications in the hydroxylation of side-chain or sterol-nucleus, and storage. Importantly, these mechanisms could impact neoplastic processes, resulting in the modulation of the microenvironment favoring cellular transformation, then tumor development.

### The role of sterol regulatory element-binding protein 2 (SREBP-2)

The activity of master regulator SREBP-2 which is key in the synthesis *de novo* of cholesterol, has been broad described in the management of low concentrations of this steroid, activating gene expression that promotes cholesterol synthesis and endocytosis, such as HMG-CoA reductase and LDL-R, respectively 66. SREBP-2 and SCAP (SREBP cleavage-activating protein) integrate a sensor of cholesterol in the endoplasmic reticulum, subtly regulating intracellular cholesterol concentrations. Moreover, LDL-R concentration in plasmatic membranes is modulated by three factors: the activity of SREBP-2, the pro-protein convertase subtilisin/kexin type 9 (PCSK9) expression and, the inducible degrader of LDL-R (IDOL) 67. Considering the critical role of LDL-R in neoplastic phenomena, specifically the regulation of PCSK9 and IDOL has not been characterized in detail under a tumoral environment.

Taking into account the intracellular cholesterol concentration could be a determinant factor for the development of neoplastic processes, elevated expression of SREBP-2 has been correlated with invasive breast carcinomas and a poor prognosis condition 68. In this sense, deletion of SREBP-2 inhibits migration and invasion phenomena in cellular models, as well as this phenomenon protects against osteolysis associated to BC 68. BC cells secrete the receptor activator of nuclear factor-κB ligand (RANKL) which promotes osteoclast formation and this activation, resulting in excessive bone resorption 69. Indeed, RANKL-CREB signaling induces the expression of SREBP-2, playing a key role in osteoclast formation; thus, this alteration promotes cytokines-release from the bone matrix, increasing BC proliferation 70, 71. These data indicate the complex relationships between these cellular processes, in which positive loops are generated among osteoclast formation and intracellular cholesterol regulation.

**Figure 2:**
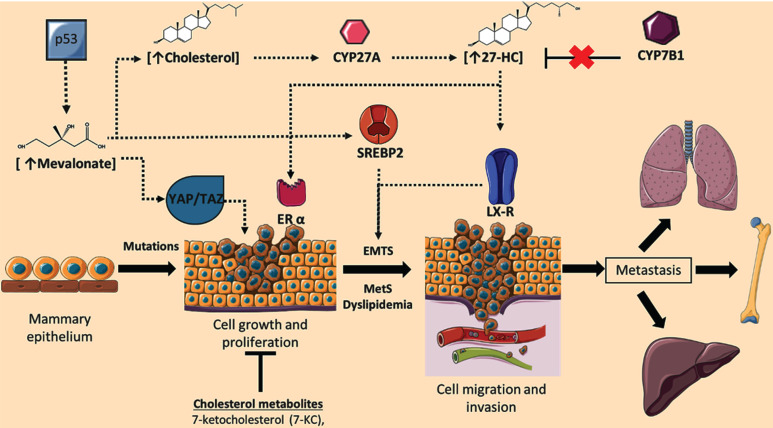
** Role of cholesterol targets and metabolites on the regulation of breast cancer (BC) progression.** During BC development, cellular concentrations of mevalonate, cholesterol, and the metabolite 27-hydroxycholesterol (27-HC) are increased; phenomenon associated with the induction of cellular proliferation, epithelial-mesenchymal transition (EMTs), invasion, and metastasis. Deregulation in 27-HC concentrations could occur through modifications in the expression of CYP27A1 and CYP7B1. Likewise, cellular targets such YAP/TAZ signaling, ERα, SREBP2, and LXR could regulate neoplastic events. Although cholesterol-derived molecules such as 7-ketocholesterol (7-KC) could show anti-tumoral functions. Adapted from: 73, 92, 100, 110-112.

Mevalonate synthesized by HMG-CoA reductase is a hub metabolite regulated by the SREBP-2 pathway, and then, further synthesis of geranyl-geranyl pyrophosphate is performed. YAP/TAZ are proto-oncogenes that promote tissue growth by induction of cell proliferation, stem cell amplification, as well as inhibition of apoptosis by expression of factor TEAD (Figure 2) 72. Critically, YAP/TAZ activity is regulated by geranyl-geranyl pyrophosphate through Rho GTPase activation. Mevalonate produced through SREBP-transcriptional activity in tumor cells is stimulated by its oncogenic cofactor mutant p53 (mutations R_175_H and R_273_H) 73. Indeed, mutant p53 in mice models is associated with metastatic tumors compared to p53 null mice 74, 75, and mutant p53 expression is correlated in preclinical cancer models with invasion, migration and metastasis 76, 77. Importantly, depletion of mutant p53 is enough to phenotypically return BC-cells to acinar-like morphology. Genome-wide analysis has identified the mevalonate pathway as an upregulated target of mutant p53 78, which could bind to sterol gene promoters via SREBP-transcription factors, inducing a high expression of sterol biosynthesis genes 78, then metabolites such as geranyl-geranyl pyrophosphate. Under these conditions, the role of dyslipidemias could exacerbate the metabolic dyshomeostasis in cancer cells and synthesis of precursor metabolites such as geranyl-geranyl pyrophosphate interrelates with non-canonical growth and proliferation signals dependent on cholesterol synthesis.

Concomitantly with SREBP-2 signaling, a contrarregulatory mechanism that responds to excessive concentrations of cholesterol has been described. Nuclear factor erythroid-derived 2-related factor 1 (Nrf1) is a direct sensor of high cholesterol concentrations wherein Nrf1 accumulates in ER, and cleavaged domain performs a function as a regulator of the Liver X receptor (LXR) and blockage of scavenger receptor (CD36) activity 79. Therefore, the modulation of these counter-regulatory mechanisms (SREBP-2 and Nrf1), and the accumulation of metabolites such as mevalonate, geranyl-geranyl pyrophosphate or cholesterol could trigger the development of neoplastic processes. In line with this notion, there is evidence suggesting the loss of Nrf1 is associated with genomic instability, resulting in the generation of micro-nuclei, abnormal mitosis, and a decrease in protein expression of targets involved in cell cycle 80. These observations suggest a critical function of Nrf1, both in the modulation of cholesterol metabolism, and a possible role on tumor development. Therefore, the subtle regulation of mechanisms mediated by SREBP2 and Nrf1 could negatively regulate metabolic and cellular processes present in tumor cells.

## Intracellular metabolism of cholesterol

Although, several metabolites accumulate by activation of the SREBP-2 pathway, a broad family of metabolites derived from cholesterol oxidation is generated by reactions performed by cytochromes, oxidation through reactive oxygen species, or by the combination of both phenomena 81, 82. For instance, oxysterols are oxidized molecules derivatives of cholesterol, associated with pro-atherogenic properties. Oxysterol formation is related to LDL-oxidation (oxLDL) due to the inflammatory cellular responses in tunica intima 83, also factors such as diet could favor the increase in plasmatic concentrations, which in turn are incorporated in plasmatic triglyceride-rich lipoproteins 84. Notwithstanding, HDL could remove oxidized lipids from the surface of oxLDL, including oxysterols such as 7-ketocholesterol and 7β-hydroxycholesterol 85.

Oxysterols are 27 carbon molecules derived from cholesterol. In this case, hydroxylation occurs in the side-chain or the sterol-nucleus. Side-chain hydroxylation generates metabolites such as 24-hydroxycholesterol (24-HC), 25-hydroxycholesterol (25-HC) and 27-hydroxycholesterol (27-HC); while hydroxylation of the sterol nucleus generates 6-hydroxycholesterol (6-HC), 7α/β-hydroxycholesterol (7α/β-HC) and 7-ketocholesterol (7-KC) 86. Moreover, oxysterols can modulate phenomena such as membrane fluidity and intracellular signaling pathways; therefore, their role has been implicated in atherosclerosis, T2D, and neurodegenerative disorders 86.

However, oxysterols and other cholesterol-metabolites such as epoxy-cholesterols have been described as the most important metabolites in the regulation of tumor development, opening new perspectives in the explanation of neoplastic phenomena. For instance, oxysterols produced by osteoblast-like MG63CM cells promote migration on MCF7 (ER+) and MDA-MB-231 (TNBC) breast cancer cells 87-89. In addition, evidence suggests oxysterols participate in the metastatic cascade modifying the pulmonary metastatic niche and contributing to the recruitment of tumor-promoting neutrophils 90. Although, oxysterols could modulate carcinogenesis and cancer progression, the precise effect of oxysterols on carcinogenesis, or cancer progression, remains to be evaluated considering the multiple effects of these metabolites on various cell lines.

### The broad role of 27-hydroxycholesterol (27-HC)

Cholesterol 27-hydroxylase (CYP27A1) is a mitochondrial resident cytochrome-P450 enzyme which performs cholesterol hydroxylation, synthesizing 27-HC 7. Conditions such as the disposal of cholesterol in mitochondria, and the expression and activity of CYP27A1 regulates the synthesis of 27-HC 91. 27-HC could bind to the estrogen receptor α (ERα), promoting the oncogenic estrogen-dependent signaling, and contributing to BC-cells proliferation 92. Although 27-HC causes a conformational change in both the ERα and ERβ 2, and ERβ is described to be expressed in both ERα+ and ERα- tumors, however a significant heterogeneity in the association of circulating 27-HC and BC risk by ERβ has not been yet identified 93. In addition, *in vivo* experimentation suggests 27-HC treatment promotes metastasis phenomena due to LXR agonist function, inducing EMTs 92, 94. Moreover, exposure to 27-HC in breast carcinoma MCF7 cells showed lower expression of E-cadherin and β-catenin, indicating a role of 27-HC during the development of EMTs 95. Elevated 27-HC concentrations have been reported in plasma and breast cancer tissues on *in vivo* models 94, 96.

Taking into account 27-HC could bind to the LXR, promoting EMTs, a condition associated with the worst prognosis in BC patients 94, 96-98, LXR activation of tumor immune cells triggers CD8+ T cells suppression activity and, inflammasome formation 94, 99. Likewise, overexpression of CYP27A1 in tumor cells and TAMs promote a tumor microenvironment rich in 27-HC, which has been associated with aggressive characteristics of ER+ in post-menopausal women 92. 27-HC also promotes cell growth and proliferation in the MCF7 cells (ER+) 100. A report of proteomics in patients with TNBC versus luminal type A breast cancer, suggests LXRα levels do not show significant differences among both molecular subtypes 101. However, a slight increase in CYP7B1 expression was found in patients with luminal type A cancer, CYP7B1 is an enzyme inducer of 27-HC catabolism. Then, the efficient degradation of 27-HC in luminal type A is considered of better prognosis in this histological subtype 101. Moreover, the analysis of CYP7B1 expression in 406 ER+ tumors *versus* 63 normal breast tissue samples (The Cancer Genome Atlas) showed a CYP7B1 diminution by 50 % in ER+ tumors compared to controls, possibly the increase of 27-HC registered in ER+ tumors is related to an alteration of 27-HC catabolism (Figure 2) 100.

Likewise, in the characterization of 22 BC specimens for oxysterol content to evaluate intra- and inter-tumor variation, authors reported significant differences of 27-HC concentrations among ER+ *versus* ER- 102. Evidence suggests the heterogeneity in 27-HC content in the cancer tissues, authors propose a possible association due to differential invasion of macrophages, fibroblast and/or adipocytes, capable of synthesizing oxysterols 102. In a recent report, serum oxysterols were determined in 58 patients with primary breast carcinoma in different tumor stages before treatment, reporting lower 27-HC levels in small tumors, in contrast with higher tumor burden 103. In a prospective study, evaluation of oxysterols-profile in 29 patients before and after treatment with Tamoxifen (Tam) and aromatase inhibitors (AI) showed increased concentrations of 27-HC under AI response and not significant changes in Tam-treatment 104. In another analysis, samples from 24 patients diagnosed with primary breast carcinoma before and after surgical tumor removal were characterized, 27-HC serum levels were lower after surgery, and patients with ERα+ tumor and treated with Tam showed a reduction of 27-HC with respect to ER- tumors 87, 105.

However, studies in humans have not been conclusive. In the evaluation of 58 patients with ERα+ BC and 18 cancer-free subjects, the tumor content of 27-HC was elevated by 2.3-fold compared to controls 100. Likewise, results of the analysis of the nested case-control study of European Investigation into Cancer and Nutrition cohort (530 incident invasive BC cases/1036 controls), suggested that higher serum 27-HC levels were associated with lower BC risk among postmenopausal women; in addition, an association between 27-HC and BC risk among premenopausal women was not registered 93. On the other hand, a report of 42 BC patients treated with atorvastatin within a phase II clinical trial, reported a decreased serum 27-HC and CYP27A1 expression in tumors; however, these changes were not directly correlated with anti-proliferative responses 106.

Another member of the oxysterol family, 25-hydroxysterol (25-HC) promoted the activation of the pro-tumoral ERα cascade in ER+ cellular models (MCF-7 and BG-1 cells) 107. In line with this notion, in gastric cancer models 25-HC promoted cell invasion associated with the upregulation of matrix metalloproteinases-expression *in vitro* and *in vivo* 104, 108. Higher serum levels of 25-HC associated with hormone receptor-positive metastatic disease have been reported 104, however, this phenomenon was not replicated by other authors in a larger population 103.

Likewise, oxysterols have multiple functions in shaping the immunological landscape, considering active cholesterol metabolism shows an essential role in promoting cancer progression. For instance, similar to cancer cells, activated T cells also undergo rapid proliferation and, therefore are dependent on elevated cholesterol metabolism. Whereas SREBP2 signaling is essential for CD8 T cell proliferation and for its effector function 109, LXR signaling negatively regulates T cell activation 109. Therefore, high levels of oxysterols in the tumor microenvironment could inhibit T cell anti-tumoral immunity via LXR activation, whereas upregulation of intrinsic cholesterol synthesis *de novo* or endocytosis in T cells could boost T-cell antitumoral functions.

### Epoxide-cholesterol pathway

**Figure 3:**
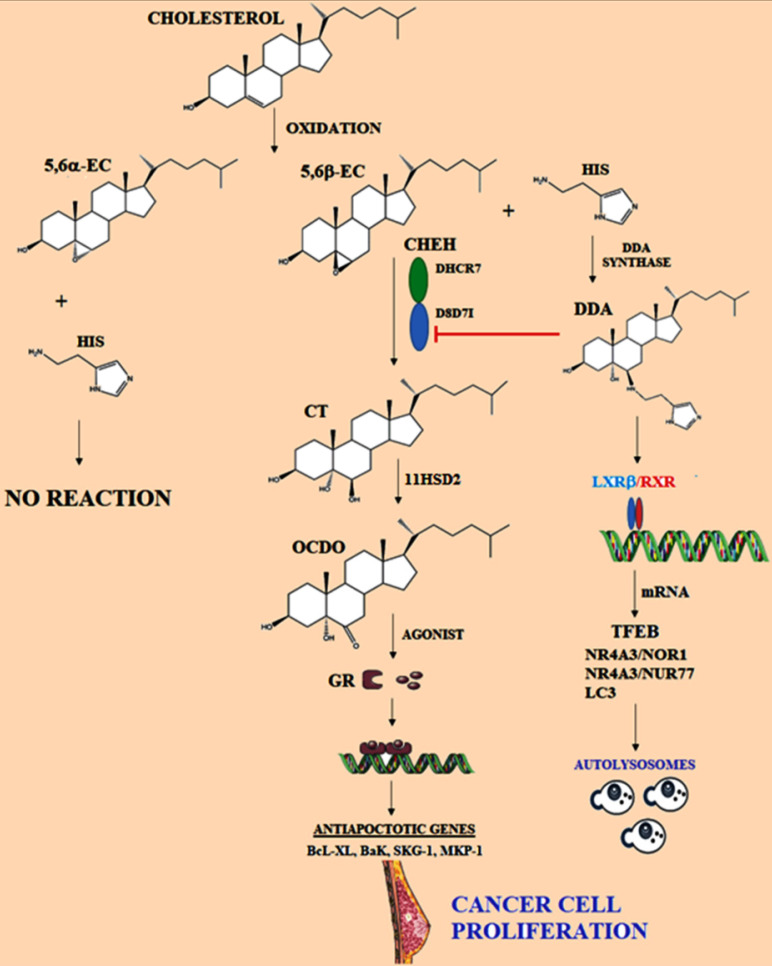
** Representation of metabolism of cholesterol epoxides**. 5,6α-EC and 5,6β-EC are metabolites produced by oxidation of cholesterol, then specifically 5,6α-EC is hydrated by Cholesterol epoxide hydrolase (ChEH), originating cholestan-3β,5α,6β-triol (CT). ChEH is a heterodimeric complex integrated by 3β-hydroxysteroid-∆8-∆7-isomerase (D8D7I) and 3β-hydroxysteroid-∆7-reductase (DHCR7). Later, CT is metabolized into 6-oxo-cholestan-3β, 5α-diol (OCDO) by 11-β-hydroxysteroid dehydrogenase of type 2 (11HSD2), OCDO is a promoter of cancer cell proliferation. OCDO is an agonist of the glucocorticoid receptor (GR) and it is translocated to the nucleus. Glucocorticoid induces the expression of genes associated with protection against cell apoptosis. Dendrogenin A (DDA) pathway is represented, which is synthesized from 5,6α-EC and histamine (His); DDA could induce autophagy in cancer cells. Adapted from: 147, 155, 157.

Other modifications occur on cholesterol structure, oxygenation reactions could be generated by enzymatic and auto-oxidation phenomena, this last process is promoted by reactive oxygen species or indirectly through lipid peroxidation 113, 114. For instance, cholesterol-5,6-epoxide (5,6-EC) is derived from cholesterol-autoxidation 115, although specifically, enzymatic systems can catalyze the synthesis of 5,6-EC 113. Tam-treatment and ERα modulators promote oxidative stress and inhibit cholesterol-5,6-epoxide hydrolases, triggering the accumulation of 5,6-ECs, these metabolites are associated with differentiation and apoptosis of BC cells 116. Evidence suggests a multifaceted role of epoxides in neoplastic pathology, being 5,6-EC the main metabolite (Figure 3). Therefore, their analysis is a critical condition.

#### 6-oxo-cholestan-3β,5α-diol (OCDO)

5,6-EC is a highly stable metabolite, allowing the synthesis of cholestane-3β,5α-triol (CT) by 5,6-epoxide hydrolase (ChEH) activity 117. In cancer cells, using CT as a substrate, the 11-β-hydroxysteroid dehydrogenase of type 2 (11HSD2) catalyzes the synthesis of 6-oxo-cholestan-3β,5α-diol (OCDO) 117, 118 (Figure 3). OCDO could promote breast tumor development through the interaction with the glucocorticoid receptor (GR) regardless of their ERα expression status, then promoting the activation of cellular-proliferation genes. Importantly, 11HSD2 is described as a regulator of BC cell proliferation both *in vitro* and *in vivo* through OCDO synthesis. Likewise, 11HSD2 is described to regulate glucocorticoid metabolism by converting active cortisol into inactive cortisone 119.

As a cortisol agonist, OCDO causes GR-translocation to the nucleus for the regulation of GR-dependent transcription 120, such as the targets cyclin-dependent kinases (CDKs), CDK4, CDK6, and cyclin D3. Although OCDO does not stimulate the transcription of SGK1 or MKP1/DUSP1 genes, which play an important role in the negative regulation of cellular proliferation, as has been reported with cortisol; however, OCDO increases MMP1 gene expression through the GR. In BC cell lines, since low OCDO concentrations (≤10 µM), treatment induces proliferation, growth, and cellular invasion, as well as tumor growth in murine models. Besides, in the absence of the GR, OCDO does not induce proliferation and invasion in cellular and *in vivo* models 104. Therefore, OCDO proliferative effects through the GR, and the identification of the enzymes regulating its synthesis could be determinant targets implicated in the biology of BC. Synthesis of glucocorticoids (GCs) is increased under stressful conditions, leading to regulation of inflammatory and immune responses, as well as cellular proliferation and apoptosis 121-124. However, the correlation between GR expression and the prognosis of breast cancer or its malignancy is still debated 125-131.

GR is considered a mediator of the GCs acute-release associated with stress conditions. *In vitro* evidence and clinical studies, suggest that stress phenomenon contributes to cancer progression 132, 133. Taking into account GR was found significantly expressed in breast cells and described to be activated by stress-induced GCs, GR might play a role in cancer development associated with stressful conditions 134. In breast cancer cells, GCs induce the expression of genes connected with protection against cell apoptosis, such as Bcl-xL, Bak, SGK-1, and MKP-1 126, 127, 135-137. Specifically, in breast cancer cell lines, GR exerts anti-apoptotic actions through the activation of NF-κB signaling 138-140. GR survival effect is mediated by its interaction with the AP-1 transcription factor family 141. Finally, GR also disrupts p53 cell survival regulation 142, as we have analyzed previously, p53 mutant is correlated in preclinical cancer models with invasion, migration and metastasis 76, 77. Therefore, the signaling pathways associated with GCs and the GR could trigger several hits in cancer development processes.

#### Cholestane-3β,5α,6β-triol (CT)

Considering 5,6-EC diastereoisomers can be hydrated by the ChEH enzyme to generate CT (Figure 3), ChEH has been reported to preferentially binds 5,6α-EC than 5,6β-EC during synthesizes of CT 143, 144. Indeed, CT was reported to be involved in carcinogenesis indirectly through the stimulation of oxidative processes 145 and thus, inhibition of ChEH may contribute to chemopreventive action of Tam and other ChEH inhibitors. Likewise, the metabolite 7-KC is described as an inhibitor of ChEH 146, then 7-KC may contribute to the accumulation of 5,6-EC. In this sense, in a 58 patient study with primary breast carcinoma, the low levels of CT were related with early stages of the disease, and a positive association was reported as dependent on the patients age. Authors have proposed a correlation among higher CT levels and a shorter disease-free survival compared with patients with lower levels 103. However, further exploration of the implications and the metabolic fate of CT during oncogenesis are necessary.

#### Dendrogenin A (DDA) pathway

On the other hand, throughout the metabolism of 5,6α-EC, the conjugation of this metabolite with histamine generates dendrogenin A (DDA), the first endogenous steroidal-alkaloid identified in mammals 147. Anti-cancer effect of DDA has been established in several mice and human cancers such as breast, melanoma, and acute myeloid leukemia 145. Indeed, treatment of cancer cells with DDA induced cancer cell re-differentiation, blockade of OCDO synthesis, and lethal autophagy in tumors 117. Evidence suggests DDA as the most potent inhibitor of ChEH, the critical enzyme for CT synthesis 8, 104, then cell differentiation and death could be induced under DDA treatment 148, 149 (Figure 3). Specifically, DDA inhibits the D8D7I subunit (3β-hydroxysterol-Δ8,7- isomerase) of ChEH leading to accumulation of 5,6-ECs 150. This accumulation shows a chemopreventive function and therapeutic effects of drugs such as Tam. Likewise, anti-cancer effects of DDA have been associated with increased tumor infiltration of T lymphocytes and CD11c+ dendritic cells. Therefore, ChEH inhibition could represent a new target involved in the anti-cancer function of DDA 147. In murine models treated with grafts of tumors B16F10 and TS/A, DDA promoted inhibition of tumor size, also increased the survival time 147. Importantly, differences of five-fold lower levels of DDA in BC tissues compared with normal matched tissues were reported 147.

On the other hand, TFEB is a master transcription factor, controlling genes involved in autophagy and lysosome biogenesis, evidence suggests LXRβ and LXRβ-agonists could act as TFEB repressors, and notably, DDA decreases LXRβ-binding to TFEB enhancer and, this phenomenon could stimulate TFEB expression 151-154 (Figure 3). Evidence revealed that LXR through DDA-binding could be a new target in autophagy and lysosome biogenesis. Reports suggest that DDA treatment in B16F10 cells increased the mRNA levels of ABCG5, NOR1, Nur77, LC3A, and LC3B 155-157, proteins involucrated in the formation of autophagosomes.

Proliferating cancer cells require high levels of cholesterol for membrane biogenesis and other functional requirements; therefore cholesterol metabolism substantially contributes to cancer progression, including cell proliferation, migration, and invasion. For instance, the cholesterol-derived metabolite 6-oxo-cholestan-3β,5α-diol, which is enriched in patients with breast cancer, binds glucocorticoid receptors and subsequently promotes tumor growth 104. In general, cholesterol metabolism substantially contributes to cancer progression, including cell proliferation, migration and invasion 158-161.

## Discussion

Cancer cells are exposed to highly fluctuating metabolic conditions, signaling metabolites, stromal biomolecules, and chemotherapeutic agents, which together generate a microenvironment that induces changes in phenotype 97, 98. Cellular adaptations may involve genetic alterations, and importantly entail transcriptional or epigenetic changes modifying cholesterol pathways 162. For instance, in the characterization of a genome-wide transcriptional survey in 995 breast tissue samples, 215 long non-coding RNAs (lncRNAs) showed a differential expression in mammary tumors compared to controls. Although several molecular processes were identified, such as activation of PI3K pathway, fibroblast growth factor, TGF-β, and activation of EGFR-dependent pathway 163, lncRNAs associated with cholesterol metabolism did not were reported for the authors 163. However, in a subsequent analysis performed by our group on these 215 lncRNA, we evidenced an altered expression of some lncRNAs associated with the lipid metabolism, such as HOTAIR and H19. In a previous report, HOTAIR could ameliorate ox-LDL-induced inflammatory response in macrophages via inhibition of NF-κB pathway 164. In contrast, silencing of H-19 could inhibit the adipogenesis and inflammation response in ox-LDL-treated macrophages 165. Possibly these lncRNAs perform critical roles in compensate inflammatory conditions on BC tissues, promoting survival. lncRNA are only one epigenetic factor that could contribute to the metabolic adaptation of tumor cells. And possibly under hypercholesterolemic conditions, several mechanisms that are performed in metabolic tissues such as hepatic or in foam cells during meta-inflammation and development of atherosclerotic plaque, are shared by breast cancer cells.

The role of cholesterol-dyslipidemias on BC risk is a phenomenon that has been strengthened, wherein the function of cholesterol-derived metabolites and alteration of cholesterol-targets induce mechanisms of cellular adaptation during BC development. For instance, bile acids, oxysterols, and epoxy-cholesterols are secondary products of cholesterol metabolism, however, in the description of these metabolites, new critical biological properties have been found, through the regulation of nuclear receptors such as LXR, FXR, ERα, ROR, GR or through G-protein coupled receptors 94, 104, 155, 166-168, which supports a new feature of these metabolites in physiological functions, and pathological disorders. Although activation of LXR, ERα, and GR promotes phenomena such as proliferation, cell invasion, and tumor growth, it is important to determine which are the cellular signals that regulate the synthesis of 27-HC, CT, OCDO, and DDA during carcinogenesis, considering the highly complex regulation of cholesterol pathways, as well as their location within the different regions of the tumor.

In addition, it is necessary characterize the mechanisms associated with dyslipidemias and their direct effects on intracellular concentration of oxysterols and epoxy-cholesterols in tumors. For instance, DDA was confirmed as a steroidal-alkaloid metabolite, identified in mammalian tissues including breast, brain, liver, spleen, and blood 147; however the diminution of DDA concentrations in tumors compared to normal tissues 147, 157 has not been explained in detail. In this case, has not been considered if the expression of the enzymes such as CHEH, 11HSD2 or DDA synthase is dependent on the SREBP2 sensor, YAP/TAZ or mutant p53. Moreover, changes in cholesterol concentration in the endoplasmic reticulum or several metabolites such as mevalonate and geranyl-geranyl pyrophosphate could be critical modulators.

In breast cancer development, extracellular cholesterol levels as well as intracellular cholesterol content should play an important role in cellular proliferation. An antagonistic role of HDL with LDL and even VLDL has been reported in the processes of cell growth, invasion, and metastasis. Thus, when intracellular cholesterol levels exceed a management threshold, the description of mechanisms that initiate and enhance processes such as EMTs, growth, migration, invasion, metastasis or resistance to chemotherapy, is a critical phenomenon that has not been elucidated in detail. The complex interactions among cholesterol pathways and the epigenetic regulation could explain several processes involved in the tumor development, as has been proposed for several lncRNAs. And how tumor cells express proteins differently to acquire various phenotypes that contribute to cellular heterogeneity in the primary tumor, possibly, the effect of cholesterol derived-metabolites could explain these phenomena in the complex cellular communication network.

Furthermore, subtle modulation between various phenotypes (EMT and MET) not only allows tumor cells to survive and proliferate in secondary sites, since by acquiring this cellular plasticity they could increase their tolerance to chemotherapeutic treatments. Moreover, this epithelial-mesenchymal plasticity that plays vital roles during normal development and tissue function 169 maintaining the cellular homeostasis, could be modified by cholesterol dyslipidemias. Therefore, regulation of this phenomenon will lead to new anti-tumoral treatments considering the cellular plasticity as a target.

Dyslipidemias and cholesterol-metabolism could impact neoplastic processes, resulting in the modulation of the microenvironment that promotes cellular oncogenic transformation, then tumor development. Indeed, the behavior of cells in the artery wall and the process of atherogenesis promoted by LDL and VLDL could share many similarities with the progression of the neoplastic processes 170. Although is not described the role of critical targets such as transporters ABCA, ABCG or SRB1 during this oncogenic transformation. Therefore, discovering of cellular mechanisms associated with cholesterol metabolism and its derivatives could facilitate the understanding of the pathology and evolution of the disease, in order to design strategies to prevent the development of breast cancer related to interventions in the lifestyle of patients with MetS, as well as in the development of effective therapies.
